# Genotyping of Rotaviruses in River Nile in Giza, Egypt

**Published:** 2020-01

**Authors:** Neveen Magdy RIZK, Abdou Kamal ALLAYEH

**Affiliations:** Environmental Virology Lab., Department of Water Pollution Research, National Research Center, Giza, Egypt

**Keywords:** Rotavirus, Genotype, Water, River Nile, Egypt

## Abstract

**Background:**

In 2013, WHO estimated the annual diarrheal mortality rate among children less than five years in Egypt was 24 deaths per 100.000, 2% was due to rotavirus infection.

**Methods:**

Eight water samples were collected monthly from the Nile water stream passing through Giza over 12 months during June 2016 to May 2017. Totally, ninety-six water samples were collected and concentrated for the detection of rotavirus group A (RV-A) using RT-PCR. Multiplex semi-nested RT-PCR was performed to identify the genotypes P and G of RV-A.

**Results:**

The detection rate of RV-A was 18.75% (18/96), whereas the rate of rotavirus genotypes G and P were 61% (11/18) and 50% (9/18), respectively. Rotaviruses G1P[8] and G1P[4] were the most common genotypes identified in our survey. In addition, the seasonal distribution findings demonstrated that the highest detection rate was 37.5% in the winter season, followed by 20.8%, 12.5% and 4% in spring, autumn and summer, respectively.

**Conclusion:**

Multiplex semi-nested RT-PCR is a useful method for rapid detection and genotyping of RV-A in surface water samples.

## Introduction

The WHO in 2010 estimated that 1.8 billion people drunk unsafe water and additionally 1.2 billion people exposed to drink contaminated water. Contaminated water was responsible for an estimated annual burden of 700.000 diarrheal deaths globally, making diarrhoea the fourth leading cause of death among young children (1). In 2008, the WHO estimated that 37% of diarrheal deaths related to rotavirus infections globally (2). During 2000–2007, the detection rate of rotavirus infections increased from 8% to 42% in Egyptian children (3). In 2013, the annual diarrheal mortality rate among children under five years in Egypt was 24 deaths per 100.000, 2% due to rotavirus infections (4).

Rotavirus is responsible for severe gastroenteritis in human. Rotavirus is ds RNA and belonging to family Reoviridae. Rotaviruses classified into G and P genotypes based on the two RNA segments nine and four. Currently, 27 G-types and 37 P-types of RV-A have been identified (5, 6). Globally, G1P[8], G2P[4], G3P[8], G4P[8] and G9P[8] are the most common genotypes causing rotavirus infections (7).

The evaluation of the incidence of G-and P-types is necessary to identify the predominant rotavirus genotype circulating in a given district that may differ between and inside the geographic areas from year to year (8). To control rotavirus incidence, vaccination, improvement of the hygiene and sanitation should be implemented (9). However, there is limited information about the prevalence of rotaviruses in surface water in Egypt. In addition, there is no well-developed surveillance system for identification of RV-A genotypes (10).

Thus, contentious surveying of rotaviruses and identifying genotypes circulating in water sources in Egypt have become necessary to assess the public health risks in Egypt particularly River Nile water (11–14). The purpose of this study was to investigate the circulating genotypes of rotaviruses in surface water samples collected from River Nile, Egypt.

## Materials and Methods

### Sample Collection

Eight water samples were collected monthly from the Nile water stream passing through Giza over 12 months during June 2016 to May 2017. Twenty liters per sample were collected and transported into our laboratory through 3 hours.

### Sample Concentration

After adding AlCl_3_ at final concentration of 0.5 mM to each water sample, the pH value adjusted to acidified pH 3.5. Each sample was filtrated using negatively charged nitrocellulose filter membrane (0.45 μm pore size, and 142 mm diameter). 75 ml of 0.05 M glycine buffer, pH 9.5 containing 3% beef extract (Lab-Limco powder, Oxoid, UK) were added for eluting the adsorbed viruses (15). An organic flocculation method was used for re-concentration (16). Finally, the pellet was dissolved in 1 ml of Na_2_HPO_4_ (0.14 N, pH 9) and kept at −70 °C until use.

### Viral RNA Extraction

RNA was extracted using BIOZOL total RNA extraction reagent (BIOFLUX—Japan) according to the manufacturer’s instructions. Briefly, after adding 500μl of BIOZOL into 100μl of each sample, the solution was incubated for 15 min on ice followed by adding 100μl of chloroform. Aqueous phase was separated and equal volume of cold isopropanol was added. The RNA pellet was formed after incubation period for 1 h at −20° C, followed by centrifugation. RNA was kept at −70°C until use.

### Detection of Rotavirus Group A using VP6

Nested RT-PCR was used for the detection of VP6 segment of rotavirus using the forward primer VP6-F primer 5′-GACGGVGCRACTACATGGT-3′ and reverse primer VP6-R 5′-GTCCAATTCATNCCTGGTG-3′ to amplify 382 bp in first RTPCR. Then, using the forward primer, VP6-NF 5′-GCTAGAAATTTTGATACA-3′ and the reverse primer, VP6-NR 5′-TCTGCAGTTTGTGAATC-3′ to amplify 155 bp (17).

### Rotavirus genotyping

Multiplex semi-nested RT-PCR was performed for genotyping of RV-A based on the characterization of VP7 and VP4 genes into G-type and P-type, respectively (17–19). The cocktail of the primers allowed to determine of G1–G6, G8–G11, P[1], and P[4]–P[11] types (Table 1).

**Table 1:** Primer sequences for genotyping of VP7 and VP4 genes of rotavirus

***Primer***	***Sequence 5′–3′***	***Sense***	***Target gene***	***Ref.***	***Primer set***	***Amplicon Length***
VP7-F	ATGTATGGTATTGAATATACCAC	+	9 (VP7)			
VP7-R	AACTTGCCACCATTTTTTCC	−	9 (VP7)	(17)	VP7-F/VP7-R	881
aBT1	CAAGTACTCAAATCAATGATGG	+	9 (VP7)		aBT1/VP7-R	618
aCT2	CAATGATATTAACACATTTTCTGTG	+	9 (VP7)		aCT2/VP7-R	521
G3	ACGAACTCAACACGAGAGG	+	9 (VP7)		G3/VP7-R	682
aDT4	CGTTTCTGGTGAGGAGTTG	+	9 (VP7)		aDT4/VP7-R	452
G8	TTRTCGCACCATTTGTGAAAT	+	9 (VP7)		G8V/P7-R	756
G9	CTTGATGTGACTAYAAATAC	+	9 (VP7)		G9/VP7-R	179
G10	ATGTCAGACTACARATACTGG	+	9 (VP7)		G10/VP7-R	266
FT5	CATGTACTCGTTGTTACGTC	−	9 (VP7)	(18)	VP7-F/FT5	729
DT6	CTAGTTCCTGTGTAGAATC	−	9 (VP7)		VP7-F/DT6	449
BT11	GTCATCAGCAATCTGAGTTGC	−	9 (VP7)		VP7-F/BT11	286
VP4-F	TATGCTCCAGTNAATTGG	+	4 (VP4)			
VP4-R	ATTGCATTTCTTTCCATAATG	−	4 (VP4)	(17)	VP4-F/VP4-R	663
2T-1	CTATTGTTAGAGGTTAGAGTC	−	4 (VP4)		VP4-F/2T-1	483
3T-1	TGTTGATTAGTTGGATTCAA	−	4 (VP4)		VP4-F/3T-1	267
1T-1D	TCTACTGGRTTRACNTGC	−	4 (VP4)		VP4-F/1T-1D	345
4T-1	TGAGACATG CAATTGGAC	−	4 (VP4)		VP4-F/4T-1	391
5T-1	ATCATAGTTAGTAGTCGG	−	4 (VP4)		VP4-F/5T-1	583
P(11)	GTAAACATCCAGAATGTG	−	4 (VP4)		VP4-F/P(11)	312
pNCDV	CGAACGCGGGGGTGGTAGTTG	+	4 (VP4)	(19)	pNCDV /VP4-R	526
pUK	GCCAGGTGTCGCATCAGAG	+	4 (VP4)		pUK /VP4-R	459
pOSU	CTTTATCGGTGGAGAATACGTCAC	+	4 (VP4)		pOSU /VP4-R	406

### Statistical analysis

The data was analyzed using one-way analysis of variation (ANOVA) in Minitab statistical program (Minitab Inc., Pennsylvania, USA). A *P-*value less than 0.05 was considered significant (20).

## Results

### Detection of Rotavirus and seasonal distribution

**Fig. 1:**
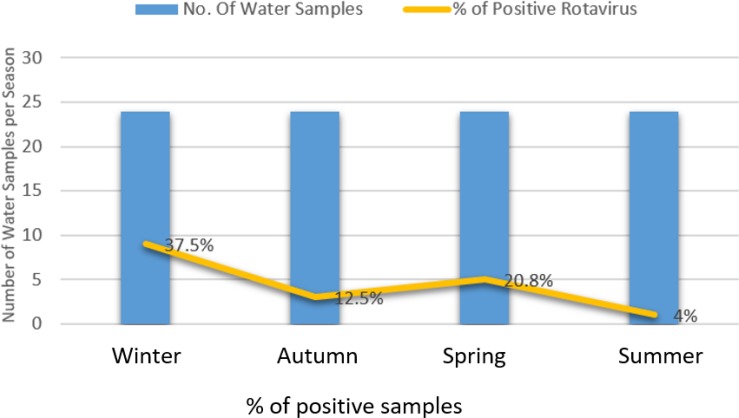
Seasonal variation of rotavirus in River Nile water

As shown in Table 2, RV-A was identified in 18.75% (18/96) of concentrated water samples using RT-PCR. The seasonal distribution showed that the highest rate of detection was 37.5% (9/24) in winter season. It was 20.8% (5/24), 12.5% (3/24) and 4.0% (1/24) in spring, autumn and summer, respectively as in (Fig. 1). Seasonal variation had no significant effect (*P*= 0.15).

**Table 2:** Detection of rotaviruses and seasonal distribution in River Nile water

***Season***	***Month***	***% of +ve Rotavirus per Month***	***% of +ve G-Type per Month***	***% of +ve P-Type per Month***	***% of +ve Rotavirus per Season***	***% of +ve G-Type per Season***	***% of +ve P-Type per Season***
Winter	Dec.	37.5% (3/8)	ND	3	37.5% (9/24)	33.3% (3/9)	44.4% (4/9)
	Jan.	37.5% (3/8)	2	1			
	Feb.	37.5% (3/8)	1	ND			
Spring	March	25.0% (2/8)	2	1	20.8% (5/24)	80.0% (4/5)	60.0% (3/5)
	April	12.5% (1/8)	1	1			
	May	25.0% (2/8)	1	1			
Summer	June	0.00% (0/8)	ND	ND	4.00% (1/24)	100% (1/1)	ND
	July	12.5% (1/8)	1	ND			
	Aug.	0.00% (0/8)	ND	ND			
Autumn	Sep.	12.5% (1/8)	1	1	12.5% (3/24)	100% (3/3)	66.6% (2/3)
	Oct.	0.00% (0/8)	ND	ND			
	Nov.	25.0% (2/8)	2	1			
Total	18.75% (18/96)		11	9	18.75% (18/96)	61.0% (11/18)	50.0% (9/18)
ND: *Not detectable*							

### Genotyping of rotavirus

**Fig. 2:**
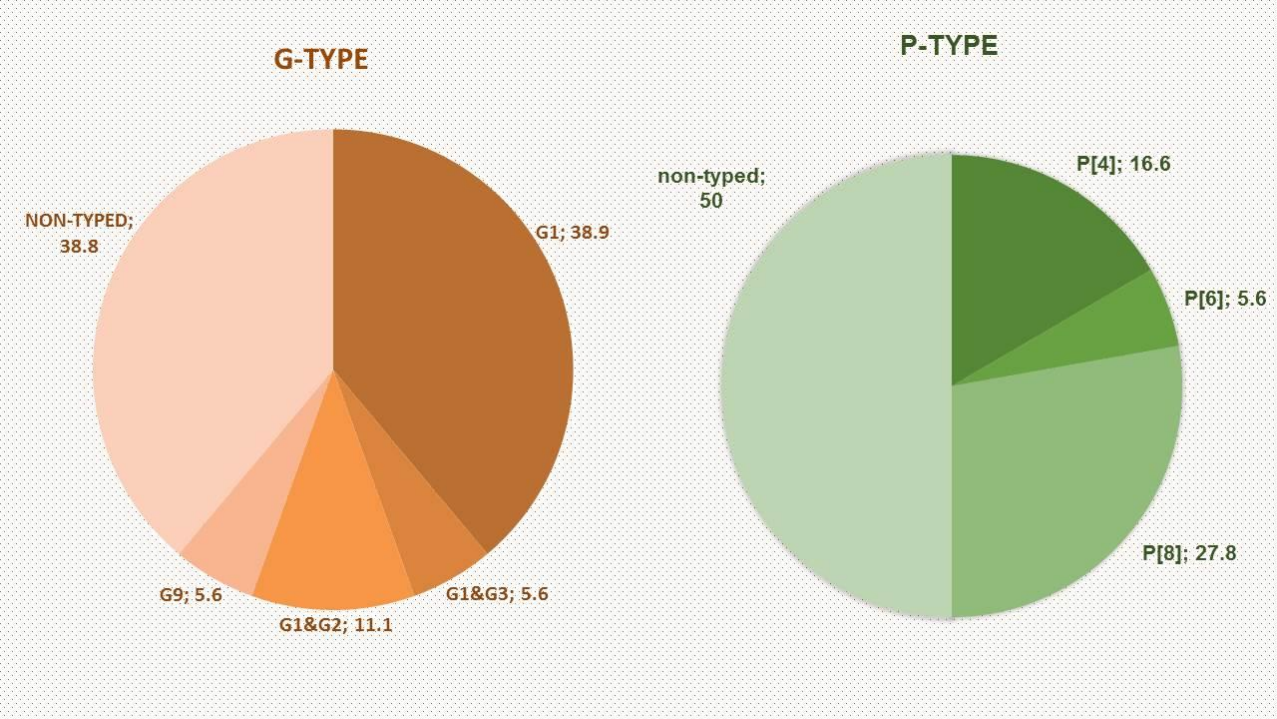
Incidence percentages of G and P genotypes in River Nile

G-type rotavirus was identified in 61% (11/18) of positive samples. G1, G2, G3 and G9 were detected using Multiplex semi-nested RT-PCR (Table 2). Co-infections of different G-types were identified in 16.7% (3/18) of positive G-type rotaviruses, (Fig. 2). No statistically significant differences were observed in seasonal distribution of G-types, (*P*= 0.344). On the other hand, 50% (9/18) of positive rotavirus were P-type rotavirus. P[4], P[6], and P[8] genotypes were identified using multiplex RT-PCR for rotavirus P-type as in (Fig. 2). No statistically significant differences in seasonal distribution of P-types was observed, (*P*= 0.270).

## Discussion

Rotavirus could be discharged into surface water resources due to its resistance to treatment processes, and their resistance may facilitate their transmission to humans and animals (21). Therefore, persistent survey of rotaviruses and identifying their genotypes are still necessary to develop the strategy of both prevention and vaccination against rotavirus. In this work, the detection rate for rotavirus was 18.75%. This overall rotavirus detection rate is relatively lower rate in comparison with those of other studies, conducted previously in water samples from Egypt in 2013 and 2015, where the most frequent detection percentages were between 20%–33% (12, 14, 22, 23).

Globally, the detection rate of rotaviruses in Thailand, Uganda and Brazil was very high more than our detection rate in Egypt, which up to 27%, 60.9% and 88%, respectively (24–29). The lower rate of rotavirus in this study might due to the concentration methods, or environmental factors like UV irradiation or sun temperature, while the high rates in Thailand, Uganda and Brazil may because of poor sanitation and cleanliness.

In this work, the rotavirus peak season is winter with rate up to 37.5%. The reason for this result is unknown but may referee to climate temperature change. In addition, detected in spring, autumn and summer with detection rate up to 20.8%, 12.5% and 4%, respectively. Our results are in agree with the rotavirus peak season of other studies conducted in Egypt and worldwide, where the most common frequent season was the winter (22, 23).

Current rotavirus immunization, authorized by the U.S. Food and Drug Administration, protected against the four-rotavirus genotypes; G1P[8], G2P[4], G3P[8], G4P[8] which are common worldwide. Two emerging combinations, G9 P[8] and G9 P[6] are becoming common (30–32). The genotypes of rotavirus are changed between socioeconomic regions of the world or even between years in a given group. Subsequently, examination of the G and P type circulation is necessary to predict the efficiency of a potential immunization for particular locale.

In this study, the genotypes of rotavirus circulating in surface water were G1 (38.9%), G2 (11.1%), G3 (5.6%) and G9 (5.6%) segments that correlated with P[8] (27.8%), P[6] (5.6%) and P[4] (16.6%). The most common frequent predominant genotypes were G1P[4] and G1P[8]. In addition, identification of G2 in our study had a significant incidence because it might be a reassortant genotype. Moreover, the co-infections G1 & G2 (11.1%) and G1 & G3 (5.6%) were also observed in the current study. These findings were partially similar to the previous studies conducted on sewage and clinical samples in Egypt, which reported that G1P[8] was usually common predominant genotype. However, our results disagreed with the same studies in its reporting about G2P[4] genotype was a common dominant genotype in Egypt (12–14). In Thailand, two genotypes (G1, and G3) were identified in river water samples, and four genotypes (G1, G2, G3, and G9) were present in irrigation canal water (24). G1 and G2 are still the most common genotypes in human, despite the fact that the conveyance of rotavirus genotypes has changed continuously and shifts in various topographical zones (33, 34). These results were in concordance with the results of the current study. Finally, the identification of the circulation of viral genotypes, particularly rotavirus, in surface water samples should support to improve vaccination and prevention guidelines against widespread rotaviruses.

## Conclusion

The presence of rotavirus in Nile water considers a public health threat. A large-scale survey in the Egyptian aquatic environment especially drinking water should be performed.

## Ethical considerations

Ethical issues (Including plagiarism, informed consent, misconduct, data fabrication and/or falsification, double publication and/or submission, redundancy, etc.) have been completely observed by the authors.
